# Chlorophyll fluorescence-based high-throughput phenotyping facilitates the genetic dissection of photosynthetic heat tolerance in African (*Oryza glaberrima*) and Asian (*Oryza sativa*) rice

**DOI:** 10.1093/jxb/erad239

**Published:** 2023-06-22

**Authors:** Jordan K Robson, John N Ferguson, Lorna McAusland, Jonathan A Atkinson, Christine Tranchant-Dubreuil, Phillipe Cubry, François Sabot, Darren M Wells, Adam H Price, Zoe A Wilson, Erik H Murchie

**Affiliations:** School of Biosciences, University of Nottingham, Sutton Bonington Campus, Loughborough, UK; School of Biosciences, University of Nottingham, Sutton Bonington Campus, Loughborough, UK; Department of Plant Sciences, University of Cambridge, Cambridge, UK; School of Life Sciences, University of Essex, Colchester, UK; School of Biosciences, University of Nottingham, Sutton Bonington Campus, Loughborough, UK; School of Biosciences, University of Nottingham, Sutton Bonington Campus, Loughborough, UK; Institut de Recherche pour le Developpement, 911 Av. Agropolis, 34394 Montpellier, France; Institut de Recherche pour le Developpement, 911 Av. Agropolis, 34394 Montpellier, France; Institut de Recherche pour le Developpement, 911 Av. Agropolis, 34394 Montpellier, France; School of Biosciences, University of Nottingham, Sutton Bonington Campus, Loughborough, UK; Institut de Recherche pour le Developpement, 911 Av. Agropolis, 34394 Montpellier, France; School of Biosciences, University of Nottingham, Sutton Bonington Campus, Loughborough, UK; School of Biosciences, University of Nottingham, Sutton Bonington Campus, Loughborough, UK; School of Biological Sciences, University of Aberdeen, Aberdeen, UK; MPI of Molecular Plant Physiology, Germany

**Keywords:** Chlorophyll fluorescence, GWAS, heat stress, photosynthesis, *Oryza glaberrima* (African rice), *Oryza sativa* (Asian rice)

## Abstract

Rising temperatures and extreme heat events threaten rice production. Half of the global population relies on rice for basic nutrition, and therefore developing heat-tolerant rice is essential. During vegetative development, reduced photosynthetic rates can limit growth and the capacity to store soluble carbohydrates. The photosystem II (PSII) complex is a particularly heat-labile component of photosynthesis. We have developed a high-throughput chlorophyll fluorescence-based screen for photosynthetic heat tolerance capable of screening hundreds of plants daily. Through measuring the response of maximum PSII efficiency to increasing temperature, this platform generates data for modelling the PSII–temperature relationship in large populations in a small amount of time. Coefficients from these models (photosynthetic heat tolerance traits) demonstrated high heritabilities across African (*Oryza glaberrima*) and Asian (*Oryza sativa*, Bengal Assam Aus Panel) rice diversity sets, highlighting valuable genetic variation accessible for breeding. Genome-wide association studies were performed across both species for these traits, representing the first documented attempt to characterize the genetic basis of photosynthetic heat tolerance in any species to date. A total of 133 candidate genes were highlighted. These were significantly enriched with genes whose predicted roles suggested influence on PSII activity and the response to stress. We discuss the most promising candidates for improving photosynthetic heat tolerance in rice.

## Teaser

A high-throughput platform for screening heat tolerance using chlorophyll fluorescence was developed and utilized to identify candidate genes underlying photosynthetic heat tolerance in African and Asian rice via GWAS.

## Introduction

The timing of heat stress events plays an important role in determining yield impacts for rice (Salvucci and Crafts-Brandner, 2004; Jagadish *et al.*, 2015; Zhen *et al.*, 2020; Li *et al.*, 2021). For example, high night-time temperatures increase rates of dark respiration, which in turn increase the consumption of photo-assimilates that may otherwise be translocated to reproductive sinks (Xu *et al.*, 2021). Daytime heat stress events can have substantial effects on productivity if they co-occur with anthesis (Lafarge *et al.*, 2016; Jagadish, 2020). During pre-anthesis developmental stages, heat stress events can detrimentally impact photosynthesis, which in turn impairs growth and the build-up of stem-stored water-soluble carbohydrates (WSC; Khan *et al.*, 2019; Qu *et al.*, 2021) that are important for subsequent grain filling. Therefore, the ability to limit pre-anthesis leaf senescence can be an important target trait for developing climatic resilience in rice.

The increasing frequency and intensity of heat stress events in key rice growing regions (Sun *et al.*, 2021; Sun *et al.* 2022) necessitates the development of novel heat-tolerant varieties. This goal can be achieved through the identification of markers that are linked to heat-tolerant loci for marker-assisted breeding, or through the identification of genes that regulate natural variation in heat tolerance that can form the basis of genetic engineering. These forward genetic approaches require the capacity to link genotypic information with phenotypic information. There is an abundant supply of single nucleotide polymorphism (SNP) genotypic datasets available for rice thanks to work such as the 3000 Rice Genomes Project (Wang *et al.*, 2018). These can facilitate genome-wide association studies (GWAS) to identify genetic regions linked to traits of interest. Consequently, the bottleneck in bridging the phenotype-to-genotype gap is the ability to quickly generate high-quality phenotypic data (Araus and Cairns, 2014; Yang *et al.*, 2020; Song *et al.*, 2021). For quantifying variation in heat tolerance this is an especially troublesome barrier to progress because the infrastructure required to expose large panels or populations of rice plants to elevated temperatures is substantial. Here, prerequisites include access to large and well-regulated controlled growth facilities or the ability to leverage field trials across geographic temperature clines.

Stabilizing photosynthesis under heat stress is an important determinant of heat tolerance, especially pre-anthesis, and it is phenotypically and genetically linked to the ability to stay green (Jagadish *et al.*, 2015; Ferguson *et al.*, 2020). Numerous aspects of photosynthesis are sensitive to increasing temperatures. For example, perturbed re-activation of Rubisco by Rubisco activase contributes to the decline in active carbon fixation with increasing temperatures (Salvucci and Crafts-Brandner, 2004; Qu *et al.*, 2021). Moreover, as temperatures increase Rubisco specificity for carboxylation compared with oxygenation declines, and therefore photorespiration increases and photosynthetic output decreases (Cavanagh *et al.*, 2022). The most heat-labile aspect of photosynthesis, however, is photosystem II (PSII) (Yamamoto, 2016; Yoshioka-Nishimura, 2018). PSII is the protein complex that catalyses the first reaction in photosynthesis. Here, a series of light-dependent electron-transfer reactions result in the splitting of water molecules, converting light energy into chemical energy (Shen, 2015). As temperatures increase beyond optimal, the manganese-stabilizing protein of the PSII complex is released, which perturbs the oxygen evolution reaction (Thompson *et al.*, 1989; Sharkey, 2005). This damage is reversible (Lydakis-Simantiris *et al.*, 1999); however, as temperatures continue to increase, PSII disassembles and there is severe denaturation of chlorophyll-containing complexes (Lípová *et al.*, 2010), representing irreparable or long term damage.

The importance of stabilized photosynthesis for facilitating heat tolerance and the integral nature of PSII to this dynamic pinpoints chlorophyll fluorescence as a technique for facilitating phenomics of heat tolerance in crop species (Ferguson *et al.*, 2021). Light energy absorbed by chlorophyll containing molecules in PSII can either facilitate photosynthesis, or be re-emitted as heat, or be re-emitted as light. The yield of re-emitted light (i.e. chlorophyll fluorescence) can be used to determine the quantum efficiency of PSII (Murchie and Lawson, 2013). In recent years, we and others have demonstrated that it is feasible to combine relatively low cost chlorophyll fluorescence platforms with custom methods of sample heating such as water baths or Peltier devices to screen the quantum efficiency of PSII in response to incrementally increasing temperatures across several species, e.g. rice (Ferguson *et al.*, 2020), tropical montane tree species (Feeley *et al.*, 2020), wheat (Coast *et al.*, 2022), and grapevine (Xu *et al.*, 2014). These data have been employed to determine parameters that quantify key aspects of the relationship between PSII efficiency and temperature. The most utilized of these is the critical temperature point (*T*_crit_), which is the temperature point at which PSII efficiency transitions from moderate to extreme reductions, and the temperature at which point PSII efficiency is 50% of its maximum, i.e. *T*_50_.

Whilst the efficiency of chlorophyll fluorescence temperature response measurements has been well demonstrated in numerous species, there have not been any demonstrated instances of this approach being utilized to screen broad intraspecific variation. Thus, demonstrating the applicability of this technique on a phenomics scale is required to understand its utility for forward genetics. To this end, we set a methodological target for this study to adapt our previous approach for screening chlorophyll fluorescence temperature responses by incorporating the use of silicone heater mats to screen substantially more samples at a time. As a test case target, we sought to screen natural variation for these responses across separate African (*O. glaberrima*; Cubry *et al.*, 2018) and Asian (*O. sativa*; Norton *et al.*, 2018) rice diversity panels. Beyond testing the utility of our new approach for screening chlorophyll fluorescence temperature responses, we sought to (i) determine the extent to which quantitative trait loci (QTL) for photosynthetic heat tolerance (PHT; e.g. *T*_crit_ and *T*_50_) were consistent between the two diverged rice species and (ii) identify novel candidate genes for PHT as targets for developing heat tolerance in rice.

## Materials and methods

### Plant material and growth conditions

Seed from all accessions comprising this study were heat treated in water at 55 ℃ for 40 min to limit fungal infections and promote germination. Seeds were sown directly into 12 litre growth containers filled with a specialized rice compost (50:50; John Innes 3: Levington M3, The Scotts Company, Ipswich, UK). Forty-eight plants were grown per container in a randomized design, representing a planting density of 0.05 plants cm^−2^. One hundred and eighty-six accessions of the Bengal Assam Asus Panel (BAAP) of *O. sativa* (Norton *et al*. 2018) and 146 accessions of *O. glaberrima* (Cubry *et al*. 2018) were grown in total (Supplementary Table S1), and the reference IR64 *O. sativa* accession was grown in each growth container.

Plants were sown and grown in a common controlled-environment growth room. Here, a combination of metal halide and incandescent lamps were lowered such that the lighting intensity measured as photosynthetically active radiation was ~550 µmol m^−2^ s^−2^ at plant level. The photoperiod was set to a 12 h day–night cycle. Temperature was set to 28 ℃ (daytime) and 25 ℃ (night-time). Relative humidity stayed within a range of 50–70%.

### Chlorophyll fluorescence–temperature response measurements

At 4 weeks post-sowing, a 4–4.5 cm portion of the third leaf of each plant was sampled. These leaf samples were arranged in a randomized manner on 2 mm-thick damp filter paper. The damp filter paper was placed on top of a 3 mm-thick aluminium sheet (40 cm×60 cm). Once all samples were arranged, a 1.5 mm-thick sheet of non-reflective glass (as described previously: Ferguson *et al.*, 2020) was placed on top of the leaf samples taking care not to disturb their positions. One hundred to one hundred and twenty samples were arranged and measured in any given *run* of measurements, where a reference map was produced on each occasion to determine the identity of each sample. Samples were collected and arranged within 45 min in a room directly adjacent to the controlled growth room.

The aluminium sheet containing the samples was subsequently placed on top of two adjacent 400 W silicone heater mats (model LM240, Thermosense, Bourne End, UK) inside of a previously described (McAusland *et al.*, 2019) custom closed chlorophyll fluorescence system (PSI, Czech Republic). The temperature of both silicone heater mats was regulated by the same proportional-integral-derivative (PID) controller (model CH102, Thermosense). Temperature feedback to the PID controller was achieved via a K-type bead thermocouple that was placed underneath the glass sheet adjacent to leaf samples, and therefore the PID controller regulated the temperature of the heater mats according to the temperature adjacent to samples on top of the filter paper. Through testing with a separate thermocouple, we determined that as long as the thermocouple regulating the PID controller was between the glass sheet and the filter paper, its specific position did not influence the temperature of 10 random points across the entire temperature-regulated area containing the samples. Further testing demonstrated the temperature at the position of the regulating thermocouple never overshot, regardless of the temperature set point.

Before measurements of chlorophyll fluorescence, samples were allowed to dark adapt for 45 min. After this point, a measuring light pulse was switched on to provide a measure of minimal chlorophyll fluorescence (*F*_o_). A follow-up saturating light pulse was used to provide a measure of maximum chlorophyll fluorescence (*F*_m_). Variable fluorescence (*F*_v_) was calculated as *F*_m_*−F*_o_ and the maximum quantum efficiency of PSII was calculated as *F*_v_/*F*_m_. Following this room temperature measurement of *F*_v_/*F*_m_, the PID controller was switched on at an initial temperature of 25 ℃. Once the set temperature was reached a timer was set for 2 min. After this 2 min period, the aforementioned chlorophyll fluorescence measurements were performed again. This was repeated at each incremental 1 ℃ of temperature up to 55 ℃, such that we obtained a value for *F*_v_/*F*_m_ at 30 temperature points and room temperature (Supplementary Video S1).

On each day of measurements, we performed one round of measurements starting with sample preparation at 09.00 h, which was typically completed around 11.00 h. A second round of measurements was then performed starting with sample preparation at 11.30 h, which was typically completed at around 13.30 h.

### Estimation of *T*_crit_, *T*_50_, *m*_1_, and *m*_2_

Raw data coming from the FluorCam 7 software used to operate the closed chlorophyll fluorescence system was quality checked and formatted within R as described previously (Ferguson *et al.*, 2020) utilizing the following packages: plyr (Wickham, 2011), reshape2 (Wickham, 2007), and ggplot2 (Wickham, 2009).

We estimated *T*_crit_, *m*_1_, and *m*_2_ via the breakpoint modelling approach we have described previously (Ferguson *et al.*, 2020) that utilizes the segmented() function in the R package segmented (Muggeo, 2017). *T*_crit_ is a computationally determined breakpoint in the relationship between *F*_v_/*F*_m_ and temperature where the response of *F*_v_/*F*_m_ transitions from a slow to a rapid decline. *m*_1_ and *m*_2_ are the slope values from linear models that define *F*_v_/*F*_m_ as a function of temperature before and after *T*_crit_ (Supplementary Fig. S1). Additionally, for this study we also estimated *T*_50_, which we define as the temperature point where *F*_v_/*F*_m_ is 50% of the maximum value estimated on a sample-by-sample basis. This was achieved first by extracting the *F*_v_/*F*_m_ value measured at 25 ℃, which was always the maximum value for *F*_v_/*F*_m_. We then constructed an inverse linear model of that used to estimate *T*_crit_, i.e. where temperature becomes the dependent variable and *F*_v_/*F*_m_ is the independent variable. We then generated a segmented model based on this linear model as described previously. Using this segmented model, we predicted the temperature (*y*), where *F*_v_/*F*_m_ (*x*) was 50% of the previously extracted maximum value.

### Statistical analyses

To account for unwanted variance with the traits of interest, we performed linear mixed models to extract genotype variance components using the lmer() function from the lme4 R package (Bates *et al.*, 2015). The models were constructed as:

$Y\;=\;Z_{ij}+\;Z_{k}+\;Z_{l}+\;e$

where *Y* represents the vector of responses (*T*_crit_, *T*_50_, *m*_1_, or *m*_2_); *Z* represents a matrix of random effects due to the interaction between round and time of measurements (*ij*), the container from which a plant originated (*k*), and the genotype (*l*); and *e* is a vector of random errors. Genotype best linear unbiased predictors (BLUPs) were extracted from these models using the ranef() function from lme4. BLUPs were added to the population mean for each trait obtained from the above-described models to generate adjusted means that thereby controlled for unexplained variance in the traits. This approach was taken for each species separately since the experiments for each were also performed separately. Unless stated otherwise, all further statistical analyses and genetic mapping were performed using BLUPs (Supplementary Table S2). The variances extracted from each linear mixed model were used to estimate broad sense heritability (*H*^2^) as the ratio of the variance due to genotype, i.e. genotypic variation, and the summation of variation from all sources, i.e. phenotypic variance.

For each species, we explored correlations between all pairwise trait interactions via Pearson’s correlation coefficient. These interactions were visualized via a network plot constructed using the corr R package. Further graphical plotting was performed using the ggplot2 R package, with some post-processing performed in Affinity Designer (Serif).

### Genome wide association mapping

Since genotypic data was separate for the two species, GWAS were carried out individually for each population using previously published pipelines. All SNP marker sets used in this study were aligned to the Nipponbare high quality reference genome (IRGSP-1.0), with bioinformatic pipelines, software and SNP filtering steps all described in more detail in Norton *et al.* (2018) and Cubry *et al.* (2018). For the *O. sativa* Bengal Assam Aus Panel (BAAP), GWAS was undertaken using PIQUE (Parallel Identification of QTLs Using EMMAX) as in Norton *et al.* (2018) and a latent factor mixed model (LFMM) was subsequently performed using the lfmm R package (Caye *et al.*, 2019), using the published 2 053 863 imputed SNP marker-set filtered for minor allele frequency (MAF) >0.05 and missing data <0.1 (Norton *et al.*, 2018). GWAS of the *O. glaberrima* population was performed via a bioinformatics pipeline utilizing GAPIT (Lipka *et al.*, 2012) and encompassing multiple models including LFMM and efficient mixed model association (EMMA), as in Cubry *et al.* (2020), using 892 539 imputed SNP markers (Cubry *et al.*, 2018) filtered for MAF >0.05 and missing data <0.05. Results from all analyses were visualized via QQ and Manhattan plots using the qqman R package (Turner, 2018). QQ plots were used to assess the two best fitting GWAS models for each trait within each population and to determine the significance threshold for SNP calling within these models. For most traits, visualizing the distribution of the GWAS *P*-values (Supplementary Figs S2–S6) demonstrated a reduction in their effect compared with what would be expected for a normal distribution, likely due to the high polygenic nature of photosynthetic heat tolerance. Therefore, we used a less stringent threshold of −log_10_(*P*-value) <4 to determine SNPs of interest in most models.

A linkage disequilibrium (LD)-based clumping procedure on PLINK (Purcell *et al.*, 2007) was used to process significant SNPs into putative QTLs based on average genome-wide LD (150 kb and 243 kb respectively in *O. glaberrima* and BAAP populations, in accordance with previously published data). To reduce the likelihood of highlighting false positives, QTLs were discarded if they contained fewer than two SNPs (Norton *et al.*, 2018).

Local LD was calculated between each SNP pair within a 500 kb region either side of each QTL peak for the BAAP population using the LDheatmap R package (Shin *et al*., 2006) to create LD heatmaps and matrices. All genes within these 1 Mb regions were annotated using the IRGSP-1.0 (International Rice Genome Sequencing Project) reference genome assembly from the Rice Annotation Project Database (RAP-DB). Genes containing at least one SNP in LD (*r*^2^>0.3) with a significant SNP from GWAS were extracted for further analysis. This list of genes were used for Gene Ontology (GO) enrichment analyses using the PANTHER classification system (Mi *et al.*, 2019). Candidate genes were shortlisted based on functional classification, GO, homology, expression over developmental stages based on information from the Rice Genome Annotation Project (RGAP), RAP-DB and RiceXPro, and differential expression within published rice heat stress transcriptomic studies (Liu *et al.*, 2020; Sharma *et al.*, 2021).

## Results

### Analysis of phenotypic variation

**Fig. 1.**
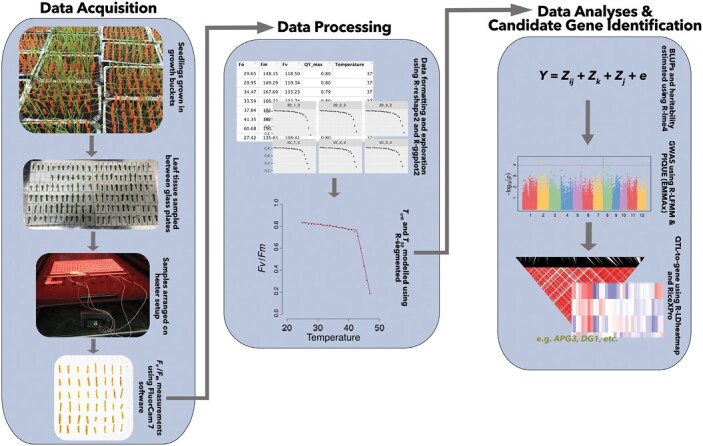
Flow diagram demonstrating steps of data acquisition, data processing, and data analysis leading to the identification of candidate genes.

**Fig. 2.**
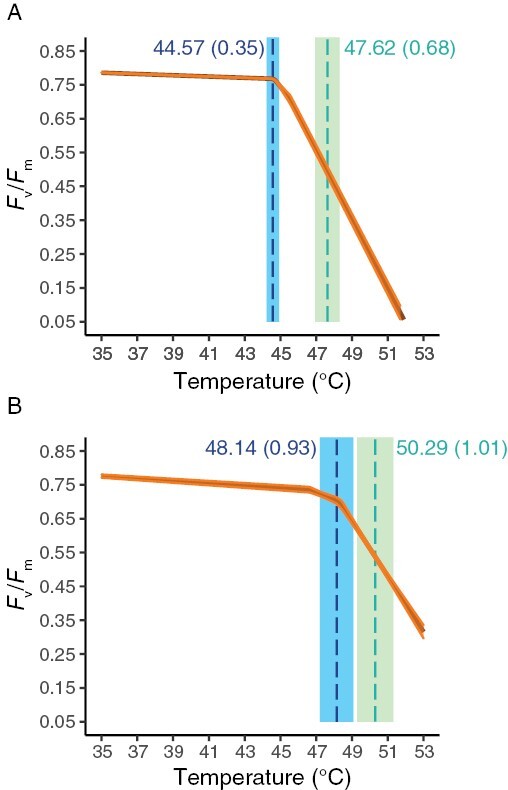
Example of segmented models fitted to two distinct *Oryza sativa* accessions. The orange solid line is the mean predicted model fit from four biological repeats, where the shaded area represents the standard error of the mean. Mean *T*_crit_ and *T*_50_ are indicated with blue and green dashed lines respectively with associated standard errors. (A) Accession IRGC_28958 (*Oryza sativa*). (B) Accession IRGC_28994 (*Oryza sativa*).

Our data acquisition and processing pipeline facilitated the generation of a dataset comprising the photosynthetic heat tolerances (PHTs) of 146 *O. glaberrima* and 186 *O. sativa* accessions within 5 weeks of sowing seeds (Fig. 1; Supplementary Table S1). Through segmented modelling, we benchmarked PHT as *T*_crit_, *T*_50_, *m*_1_, and *m*_2_ as described previously (Ferguson *et al*., 2020; Supplementary Fig. S1), thereby characterizing the whole response of *F*_v_/*F*_m_ to rapidly increasing temperatures (Fig. 2).

**Fig. 3.**
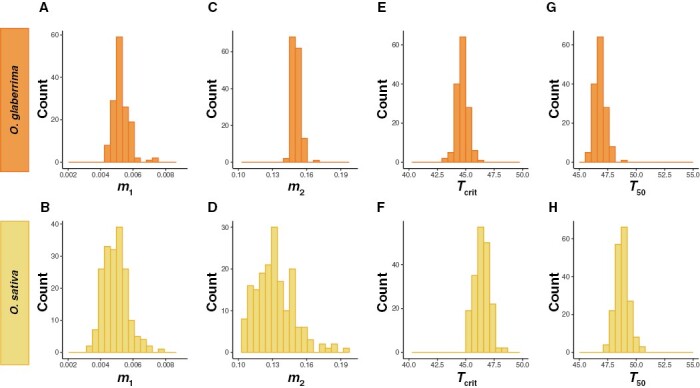
Natural variation for all parameters modelled from the segmented relationship between *F*_v_/*F*_m_ and temperature. (A, B) *m*_1_ for *O. glaberrima* and *O. sativa*, respectively. (C, D) *m*_2_ for *O. glaberrima* and *O. sativa*, respectively. (E, F) *T*_crit_ for *O. glaberrima* and *O. sativa*, respectively. (G, H) *T*_50_ for *O. glaberrima* and *O. sativa*, respectively.

In general, the intraspecific variation for PHT within *O. sativa* was greater than the intraspecific variation within *O. glaberrima* (Fig. 3). For example, *m*_2_ varied from 0.142 to 0.169 in *O. glaberrima* (Fig. 3C) and from 0.092 to 0.206 in *O. sativa* (Fig. 3D). Similarly, *T*_crit_ varied from 45.7 to 48.8 in *O. glaberrima* (Fig. 3E) and from 47.3 to 50.7 in *O. sativa* (Fig. 3F).

The variation in PHT within *O. sativa* was shifted towards reduced sensitivity to temperature compared with *O. glaberrima*. For example, the population means for the slope metrics (*m*_1_ and *m*_2_) were greater in *O. glaberrima* (0.0053 and 0.151, respectively) compared with *O. sativa* (0.0049 and 0.132, respectively; Fig. 3A–D), where reduced values here indicate a less extreme response. Similarly, the range in *T*_crit_ and *T*_50_ values and their associated population means were reduced in *O. glaberrima* (44.7 and 46.8) compared with *O. sativa* (46.4 and 48.8; Fig. 3E–H), where greater values here indicate a less sensitive response to temperature, i.e. *F*_v_/*F*_m_ reaches the critical temperature point and 50% of the maximum *F*_v_/*F*_m_ at a higher temperature in general in *O. sativa*.

Except for the *m*_2_ parameter estimated in *O. glaberrima*, for which our mixed effect model did not well explain the data (*R*^2^=0.18), all of the PHT metrics estimated across both species demonstrated moderate-to-high broad sense heritabilities considering their complex nature (*H*^2^; Table 1). The most heritable trait was *T*_crit_, which was estimated at 0.65 in *O. sativa* and 0.61 in *O. glaberrima*. The least heritable trait for *O. glaberrima* was *m*_2_ (0.09), which conversely had a moderate heritability of 0.53 in *O. sativa*. The least heritable trait for *O. sativa* was *m*_1_ (0.48), which was also much less heritable in *O. glaberrima* (0.27).

**Table 1.** The population mean, broad sense heritability (*H*^2^), and goodness of fit of the linear mixed model (*R*^2^) for each trait measured on each species.

Trait	Population mean	*H* ^2^	*R* ^2^
*O. glaberrima*			
* T*_50_	46.847	0.53	0.76
* T*_crit_	44.709	0.61	0.83
* m*_1_	−0.005	0.27	0.42
* m*_2_	−0.151	0.09	0.18
*O. sativa*			
* T*_50_	48.763	0.61	0.74
* T*_crit_	46.415	0.65	0.72
* m*_1_	−0.005	0.48	0.49
* m*_2_	−0.132	0.53	0.52

The only common correlation for both species was the strong positive correlation between *T*_crit_ and *T*_50_, suggesting that genotypes that transition to the *m*_2_ phase of the association between temperature and *F*_v_/*F*_m_ fastest reach 50% of maximum *F*_v_/*F*_m_ at the lowest temperatures, i.e. reduced PHT (Supplementary Table S3). The correlation between the *T*_crit_ parameter and the *m*_2_ parameter was significant for both species, but the direction of the correlation was reversed. Here, these parameters shared a positive correlation across the *O. sativa* accessions, but negative across the *O. glaberrima* accessions. This suggests that *O. sativa* accessions that transition to the *m*_2_ phase at the lowest temperature demonstrate the lowest rate of decline in *F*_v_/*F*_m_ from that point onward, whereas it suggests the opposite for the *O. glaberrima* accessions.

For *O. glaberrima* the only additionally significant correlation was the positive correlation between *m*_1_ and *T*_50_, suggesting that *O. glaberrima* accessions with the fastest initial decline in *F*_v_/*F*_m_ reach 50% of the maximum *F*_v_/*F*_m_ at the lowest temperature. This is also reflected in *m*_1_ and *T*_crit_ showing a marginally non-significant (*P*=0.06) positive correlation also (Supplementary Table S3). For *O. sativa*, significant positive correlations were detected between *T*_crit_ and *m*_1_ and between *m*_1_ and *m*_2_ (Supplementary Table S3), which suggests that lines that respond most strongly to the initial temperature increases (i) transition to the *m*_2_ phase quickest and (ii) also have the fastest rates of decline in *F*_v_/*F*_m_ after the transition. Finally, *m*_2_ and *T*_50_ demonstrated a significant negative correlation across the *O. sativa* accessions (Supplementary Table S3), which suggests that lines that have the fastest rate of decline following the *T*_crit_ point reach 50% of maximum *F*_v_/*F*_m_ at the lowest temperatures.

### Genome-wide association mapping

The *T*_50_ and *T*_crit_ parameters demonstrated the highest heritabilities across the two species and were phenotypically linked to *m*_1_ and *m*_2_ in the majority (Table 1; Supplementary Table S3), and consequently we focused on these traits for our GWAS.

Marker-trait associations were tested using at least two different models, EMMA (Kang *et al.*, 2008) and LFMM (Frichot *et al.*, 2013), with additional computation of other GAPIT models including FarmCPU in the *O. glaberrima* population. For each trait within the two populations, we determined the best-fit model based on observations of the QQ plots, which describe the distribution of the *P*-values associated with all SNPs against what would be expected of a normal distribution (Supplementary Figs S2–S5). Within the *O. sativa* population there was little difference between the QQ plots, and therefore EMMA was used as the best-fit for both *T*_50_ and *T*_crit_, whereas in *O. glaberrima* LFMM was superior for *T*_50_ whilst FarmCPU fit *T*_crit_ marginally better than EMMA. QQ plots were further used to determine a cut-off significance threshold for SNPs. These plots suggested that *T*_50_ and *T*_crit_ were polygenic in both species (Supplementary Figs S2–S5) and that a stringent significance threshold would be inappropriate for identifying SNPs of interest. Thus, to identify QTLs we employed a threshold of −log_10_(*P*)>4 in all but one of the models (*T*_50_-Glab-LFMM; Supplementary Table S4).

**Fig. 4.**
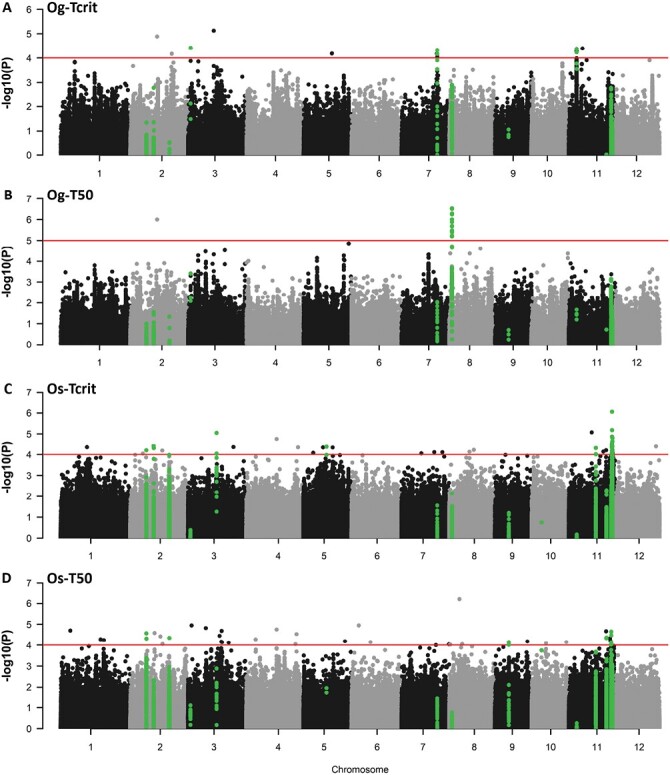
Manhattan plots for genome wide association of PHT traits within the two rice populations. (A) *T*_crit_ association with *O. glaberrima* (Og) SNPs according to FarmCPU GWAS model; (B) T50-Og association according to LFMM model; (C) *T*_crit_ association with *O. sativa* (Os) according to EMMA model; (D) T50-Os association according to EMMA model. Solid red line indicates of suggestive SNP significance threshold based upon polygenicity assessment of QQ plots. SNPs within the identified QTLs are highlighted in green to show distinct distribution across traits and populations.

Significant SNPs were clumped into putative QTLs containing two or more significant SNPs based on global LD of 150 kb in *O. glaberrima* (Cubry *et al.*, 2018) and 243 kb within the *O. sativa* BAAP population (Norton *et al.*, 2018). Through comparison of the best-fitting GWAS models, 15 distinct QTLs were identified within the *O. sativa* and *O. glaberrima* populations (Table 2; Fig. 4), with high consensus in SNPs within these regions between the two best-fitting models for each trait. Whilst there were no overlapping QTL regions between the two species, there was overlap between traits within the *O. sativa* population. For example, within both of the *T*_50_ QTLs on chromosome 2 (Os-T50-2a and Os-T50-2b) and within Os-T50-11a, a singular significant SNP was also identified for *T*_crit_. Likewise, a significant *T*_50_ association was highlighted in Os-Tcrit-11a (Fig. 4).

**Table 2.** Location of putative QTLs identified from GWAS of *T*_50_ and *T*_crit_ traits within the Bengal Assam Aus sub-population of *Oryza sativa* (BAAP) and sub-population of *Oryza glaberrima* (Glab).

QTL-ID	QTL location			Number of significant SNPs			
	Chr	Range	Peak (Mb)	*T*_50_-BAAP	*T*_crit_-BAAP	*T*_50_-Glab	*T*_crit_-Glab
Os-T50-2a	2	10.06–10.14	10.059	2 ^ab^	1 ^a^		
Os-Tcrit-2	2	14.57–14.71	14.568		3 ^a^		
Os-T50-2b	2	24.29–24.38	24.382	2 ^a^	1 ^b^		
Og-Tcrit-3	3	1.61–1.61	1.61				2 ^a^
Os-Tcrit-3	3	17.89–17.89	17.894		3 ^a^		
Os-Tcrit-5	5	14.45–14.45	14.452		2 ^b^		
Og-Tcrit-7	7	22.24–22.26	22.256				3 ^a^
Og-T50-8	8	1.71–1.78	1.757			20 ^a^	
Os-T50-9	9	8.76–8.76	8.757	2 ^a^			
Os-T50-10	10	6.25–6.25	6.255	2 ^b^			
Og-Tcrit-11	11	4.96–4.97	4.964				2 ^a^
Os-Tcrit-11a	11	16.88–16.89	16.88	1 ^b^	2 ^ab^		
Os-T50-11a	11	23.25–23.51	23.25	3 ^a^	1 ^a^		
Os-T50-11b	11	26.40–26.48	26.456	12 ^a^			
Os-Tcrit-11b	11	26.81–27.25	26.978		40 ^b^		

Maximum number of significant SNPs within the QTL is reported according to the best (a) and second best (b) fit GWAS model.

Whilst most of the QTLs identified from this PHT screen appear to be novel, there are a couple that overlap with previously identified heat-tolerance QTLs. The BAAP population-specific *T*_crit_ QTLs on chromosome 3 (Os-Tcrit-3) and chromosome 5 (Os-Tcrit-5) overlap respectively with slpc3.1, shoot length under heat stress (Kilasi *et al.*, 2018), and qhts-5, spikelet fertility under heat (Ishimaru *et al.*, 2016). Also of note with respect to *O. glaberrima* is the *T*_50_ QTL on chromosome 8 (Og-T50-8), which is just 236 kb from a QTL identified in environmental GWAS by Cubry *et al.* (2020) for BIOCLIMATIC PRINCIPLE COMPONENT 2, which is explained primarily by the mean temperatures of the driest and coldest quarters. As the *O. glaberrima* SNP dataset was generated using alignment to the *O. sativa* Nipponbare reference genome (Cubry *et al.*, 2018), the region between the two QTLs was investigated using the NCBI database. This identified 31 genetic loci, 14 of which have orthologues in *O. glaberrima* (Supplementary Table S5). These genes include those with roles in heat tolerance (Os08g0135900) and reactive oxygen species (ROS) homeostasis (Os08g0133000 and Os08g0133700), as we discuss later.

**Fig. 5.**
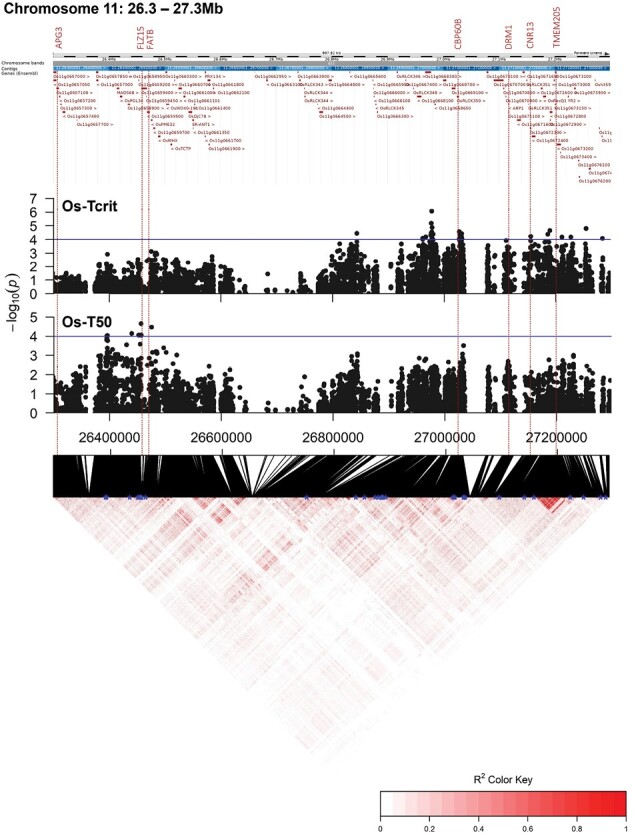
Precise mapping of 1 Mb region (26.3–27.3 Mb) on Chromosome 11. Zoomed Manhattan plots showing SNP associations with *T*_crit_ and *T*_50_ in the *O. sativa* population are plotted against a linkage disequilibrium (LD) heatmap, with blue asterisks highlighting significant SNPs (*P*<0.0001). All genes within the region are further plotted, with dotted lines highlighting the positions of select PHT candidate genes (Table 3) within LD (*r*^2^>0.3) of significant SNPs.

**Fig. 6.**
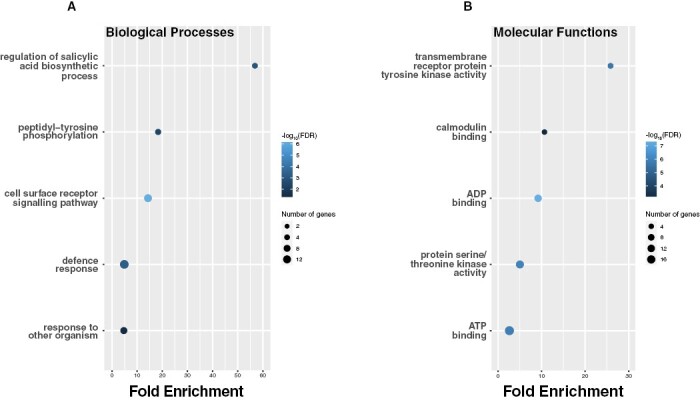
Gene ontology (GO) enrichment analyses. (A) Significantly enriched biological process terms within the identified gene list. (B) Significantly enriched molecular function terms within the identified gene list.

Since the genome is better annotated for *Oryza sativa*, and the BAAP population has been selected specifically for its increased abiotic stress resources, we performed a more detailed downstream bioinformatics analysis of all the QTLs identified within the BAAP population. Local linkage disequilibrium (LD) around each QTL was calculated to identify genes co-localizing with the significant SNPs (Fig. 5). This approach identified 133 genes within LD (*r*^2^>0.3) of significant SNPs (Supplementary Table S6). We performed GO enrichment analyses to benchmark the likelihood of these genes being involved in PHT. Here, we tested whether these 133 genes were significantly enriched for GO terms associated with biological processes, molecular functions, and cellular components. No GO cellular component terms were identified as significantly enriched in this set of genes, but terms for biological processes and molecular processes were enriched compared with what would be expected according to how many genes within the rice genome represent those terms (Fig. 6; Supplementary Table S7). For biological processes, five granular (specific) terms were enriched: ‘regulation of salicylic acid biosynthetic processes’, ‘peptidyl-tyrosine phosphorylation’, ‘cell surface receptor signalling pathway’, ‘defence response’, and ‘response to other organism’ (Fig. 6A). Five granular terms were also enriched for molecular functions: ‘transmembrane receptor protein kinase activity’, ‘calmodulin binding’, ‘ADP binding’, ‘protein serine/threonine kinase activity’, and ‘ATP binding’ (Fig. 6B).

To pinpoint loci that might underlie photosynthetic heat tolerance we analysed the functional annotation, GO terms and literature associated with each of these genes alongside available RNA-Seq data showing transcriptomic changes in response to heat. We found that 19 of these genes are differentially expressed in response to heat in either IR64 or Annapurna seedlings according to a previously published RNA-Seq analysis (Sharma *et al.*, 2021) and 11 genes are expressed in the chloroplast according to GO annotation of cellular compartment (Supplementary Table S6). Eleven of the genes, or their homologues, reportedly have a function in stress response, photosynthesis or carbon partitioning, chloroplast development, stomatal density, or senescence according to a literature search. Taken together, this generated a shortlist of 30 genes (Table 3) that are strong candidates for the QTLs identified within this study.

**Table 3.** Candidate genes underlying QTLs from BAAP population with relevant functions, according to literature analysis, differentially expressed genes (DEG) showing a more than 2-fold change in response to 37/42 °C heat in IR64/Annapurna seedlings according to RNA-seq (Sharma *et al.*, 2021) and chloroplast (Chl.) localization according to Gene Ontology (GO) analysis of cellular compartment.

Loci (QTL)	Name	Description	Function	DEG	Chl.	Reference
Os02g0274900 (Os-T50-2a)	*GMST1*	Golgi localized monosaccharide transporter 1	Photosynthesis and carbon partitioning (Arabidopsis)	—	Yes	Cho *et al.* (2011)
Os02g0275200 (Os-T50-2a)	*FUT1*	Galactoside 2-α-l-fucosyltransferase	—	Down	—	
Os02g0276200 (Os-T50-2a)	—	Probable inactive nicotinamidase At3g16190	—	Up	—	
Os02g0448400 (Os-Tcrit-2)	*SYN2*	Synaptotagmin-2	Heat stress (Arabidopsis)	—	—	Yan *et al.* (2017)
Os02g0448600 (Os-Tcrit-2)	—	Pentatricopeptide (PPR) repeat-containing protein-like At4g22758	—	—	Yes	
Os05g0311500 (Os-Tcrit-5)	*LOR8*	Protein LURP-one-related 8	—	Down	—	
Os05g0311801 (Os-Tcrit-5)	*IPT3*	Adenylate isopentenyltransferase 3	—	—	Yes	
Os05g0312000 (Os-Tcrit-5)	*SPL40*	Mediator of RNA polymerase II transcription subunit 33A	Photosynthesis and disease resistance (rice)	Up	—	Sathe *et al.* (2019)
Os05g0312600 (Os-Tcrit-5)	*CML21*	Calmodulin-like protein 21	Abiotic stress response (grapevine)	—	Yes	Aleynova *et al.* (2020)
Os05g0314700 (Os-Tcrit-5)	—	Uncharacterized protein	—	Up	—	
Os05g0315100 (Os-Tcrit-5)	*OsDG1*	Delayed greening 1	Chloroplast development (Arabidopsis) and stress response (rice)	Up	Yes	Chi *et al.* (2008); Chen *et al.* (2018)
Os05g0316100 (Os-Tcrit-5)	—	Putative zinc transporter At3g08650	—	Up	—	
Os05g0316200 (Os-Tcrit-5)	—	Protein SSUH2 homolog	—	Up	—	
Os05g0317200 (Os-Tcrit-5)	*LACS8*	Long chain acyl-CoA synthetase 8	—	—	Yes	
Os05g0318300 (Os-Tcrit-5)	*RNC3*	Chloroplast mini-ribonuclease III At1g55140	Chlorophyll accumulation (Arabidopsis)	Down	Yes	Hotto *et al.* (2015)
Os05g0317700 (Os-Tcrit-5)	*OsANX2*	Receptor-like protein kinase FERONIA	—	Down	—	
Os05g0318600 (Os-Tcrit-5)	-—	LOW QUALITY PROTEIN: receptor-like protein kinase FERONIA	—	Down	—	
Os05g0320800 (Os-Tcrit-5)	—	Uncharacterized protein	—	Down	—	
Os05g0321900 (Os-Tcrit-5)	*WRKY55*	WRKY transcription factor 55	Leaf senescence & defence (Arabidopsis)	—	—	Y. Wang *et al.* (2020)
Os11g0484500 (Os-Tcrit-11a)	*PGD2*	6-Phosphogluconate dehydrogenase, decarboxylating 2	—	—	Yes	
Os11g0603200 (Os-T50-11a)	*ABCF5*	ABC transporter F family member 5	—	Down	Yes	
Os11g0657100 (Os-T50-11b)	*APG3*	Albino or pale green 3	Chloroplast development (Arabidopsis)	—	Yes	Satou *et al.* (2014)
Os11g0659200 (Os-T50-11b)	*FLZ15*	FCS-Like zinc finger 15	—	Up	Yes	
Os11g0659500 (Os-T50-11b)	*FATB*	Palmitoyl-acyl carrier protein thioesterase	—	—	Yes	
Os11g0669100 (Os-Tcrit-11b)	*CBP60B*	Calmodulin-binding protein 60 B	—	Down	—	
Os11g0670900 (Os-Tcrit-11b)	—	Uncharacterized protein	—	Up	—	
Os11g0671000 (Os-Tcrit-11b)	*DRM1*	Dormancy-associated protein 1	Heat shock (*Brassica*)	—	—	Lee *et al.* (2013)
Os11g0672300 (Os-Tcrit-11b)	*CNR13*	Cell number regulator 13	Stomatal density (maize)	Up	—	Rosa *et al.* (2017)
Os11g0673100 (Os-Tcrit-11b)	*TMEM205*	Transmembrane protein 205	—	Up	—	
Os11g0678000 (Os-Tcrit-11b)	*SIS8*	Probable serine/threonine-protein kinase, sugar insensitive 8	—	Down	—	

## Discussion

### Heritable variation in photosynthetic heat tolerance highlights the development of a new breeding tool

Performing large-scale screening of heat tolerance in any crop is hampered by numerous logistical issues relating to space to grow plants and infrastructure to elevate temperatures both in controlled and in field environments (Sharma *et al.*, 2017; Ruiz-Vera *et al.*, 2018). Consequently, there is a strong requirement to develop platforms that bypass these hurdles and facilitate the rapid generation of data relating to heat tolerance. Chlorophyll fluorescence techniques are rapid and can provide information on the efficiency of particularly heat-labile components of photosynthesis that are important for defining growth and productivity (Maxwell and Johnson, 2000; Murchie and Lawson, 2013). Furthermore, it has been demonstrated that measuring various different aspects of photosynthesis on excised leaves via chlorophyll fluorescence is strongly representative of measuring the same parameter on leaves still attached to the plant in numerous crop species (McAusland *et al*., 2019; Ferguson *et al*., 2023, Preprint). This therefore opens up the opportunity to utilize chlorophyll fluorescence as a platform for rapidly screening heat tolerance. In our previous work, we have shown that *T*_crit_ and *m*_1_ as measured on excised leaf segments from rice seedlings are able to forecast adult vegetative heat tolerance measured as stay green (Ferguson *et al*., 2020), which is a common breeding-based method of scoring abiotic stress tolerance (Jagadish et al., 2015). Although effective, this previous approach suffered from throughput limitations. With the present study, these limitations were resolved by developing a heating system using silicone heater mats instead of relying on a water bath system.

Using this system, we detected significant genetic variation for PHT metrics (Fig. 3). Moreover, the broad sense heritability of these metrics were high (Table 1), especially compared with studies that have measured similar chlorophyll fluorescence parameters across diversity in other species, where heritabilities tend to be much lower (Čepl *et al.*, 2016; Herritt *et al.*, 2018; Burgess *et al.*, 2020, Preprint; Herritt and Fritschi, 2020). Indeed the heritabilities we observed are much more similar to those observed in a precisely controlled phenomics platform designed for measuring chlorophyll fluorescence in Arabidopsis (Flood *et al.*, 2016). This suggests that our phenotyping platform limits environmental noise that may confound our measurements and highlights the existence of genetic mechanisms underlying the observed variation in both species. These are attractive features of a phenotyping platform and suggest that it could provide cost-free, repeatable, and potentially valuable data to use as covariates in selection models for rice breeding. Breeding for yield while also considering information relating to heat tolerance has the potential to enhance the climatic resilience of future, highly productive rice varieties.

The main coefficients obtained from the segmented modelling used to characterize the *F*_v_/*F*_m_ temperature response, i.e. *T*_crit_ and *T*_50_ (Fig. 2), demonstrated strong positive correlations (Supplementary Table S3). However, the correlations were not perfect (Supplementary Table S3; *R*^2^=0.73 and 0.65 in *O. glaberrima* and *O. sativa*, respectively). Therefore, the aspect(s) of the response of PSII to incrementally increasing temperatures that they are characterizing are different. This is valuable for gene identification, because it allows us to detect unique QTLs underlying the different traits, even though they are positively correlated. This is evidenced by our results, for example mapping for *T*_crit_ and *T*_50_ can pick up colocalizing QTLs (e.g. on chromosome 2 and 11 in *O. sativa*; Figs 4, 5), but occasionally genetic regions only appear important for regulating one of these traits within a species (e.g. on chromosome 8 and 11 in *O. glaberrima*; Fig. 4).

In general, our data suggest that our Assam Aus diversity set of *O. sativa* is more heat tolerant than the surveyed *O. glaberrima* lines (Fig. 3). It is also interesting to note the differences in correlations between PHT parameters across the species. We have previously discussed in detail what these parameters reflect in terms of PSII activity and its response to heat stress (Ferguson *et al.*, 2020). Here, we note in particular that *T*_crit_ and *m*_2_ are positively correlated in *O. sativa* but negatively correlated in *O. glaberrima* (Supplementary Table S2). *m*_2_ describes the relationship between *F*_v_/*F*_m_ and temperature after the point (*T*_crit_) where it transitions to a rapid decline and refers more to heat *resistance* than *tolerance* in that it gauges the capacity to restrain permanent damage as opposed to maintaining typical plant function, i.e. *tolerating* high temperatures (Thompson *et al.*, 1989; Zhang and Sharkey, 2009; Ferguson *et al.*, 2020). The negative correlation between *T*_crit_ and *m*_2_ in *O. glaberrima* appears initially more logical since it suggests that lines that transition to the *m*_2_ phase faster, i.e. have reduced *T*_crit_, have a faster rate of PSII disassembly as well. This is indicative of *O. glaberrima* genotypes with high heat tolerance also having high heat resistance (reduced rate of decline of *F*_v_/*F*_m_ in the secondary temperature range after *T*_crit_). The positive correlation between these parameters in *O. sativa* would suggest the opposite. This highlights uncoupling in *O. sativa* between the tolerance of PSII to heat, which is likely conferred through mechanisms related to the capacity of the thylakoid membranes to unfold for PSII repair (Theis and Schroda, 2016), and its resistance to heat after the transition to the point where PSII deconstruction begins to take place. The underlying mechanisms that confer potential trade-offs here are of interest and could help guide target traits for crop improvement depending on the environment being selected for, e.g. mild or extreme heat stress.

### GWAS for photosynthetic heat tolerance identifies genes enriched with predicted functions associated with regulating PSII activity

Through GWAS, we have identified novel and distinct QTLs underlying PHT in diverse rice populations (Figs 4, 5; Table 2). Three times as many *T*_crit_ and *T*_50_ QTLs were identified for *O. sativa* than for *O. glaberrima*. This reflects our observation that heritability for all PHT traits was higher in *O. sativa* than in *O. glaberrima* (Table 1) and that *O. sativa* was in general more tolerant to heat stress, with higher population means for *T*_crit_ and *T*_50_ (Fig. 3). Taken together, these findings suggest that selection strength for PHT may have been reduced in *O. glaberrima* or that it harbours fewer, but of stronger effect, PHT-associated genes compared with the Asian species. Compared with other types of *O. sativa*, the *aus* varieties are considered to be highly stress tolerant. This may be a consequence of *aus* cultivars originating predominantly from Bangladesh and India (Ali *et al.*, 2011) since there appears to have been strong selective pressure on rice cultivated in the stress-prone Bangladesh and adjacent regions to be more resilient to environmental stresses (Bin Rahman and Zhang, 2018). This increased PHT is unlikely to be representative of the *O. sativa* species as a whole. The *O. glaberrima* accessions were selected from ranges of temperature, rainfall, and altitudes across western Africa, but it is unclear how limited the variation in this region might be compared with that across Asia (Cowling *et al*. 2021). Regardless, it seems that PHT mechanisms are divergent between the two populations sampled as we found no overlapping QTLs.

The enriched GO terms within the candidate genes highlight the utility of our phenotyping and GWAS approach. The biological processes and molecular functions associated with these terms pinpoint roles for these genes in PSII activity and the response to stress (Fig. 6). The role of PSII in the conversion of ADP to ATP by non-cyclic photophosphorylation (Arnon, 1984) is reflected in the enrichment of genes associated with the ‘ADP binding’ molecular function GO term. Additionally, the enriched GO terms associated with tyrosine activity (i.e. ‘peptidyl-tyrosine phosphorylation’ and ‘transmembrane receptor protein tyrosine kinase activity’) further highlight a role in PSII activity for many of the candidate genes, as tyrosine phosphorylation involves the transfer of a phosphate away from ATP (Mühlenbeck *et al.*, 2021), which may in turn increase the demand for ATP, thereby influencing PSII activity. Indeed, the increasing demand for ATP may be a result of the heat shock damage to PSII, since ATP is demonstrated to be the driving force in the repair of PSII during photoinhibition (Murata and Nishiyama, 2018). Here, ATP-dependent regulation of PSII repair under environmental stress is associated with synthesis of the D1 protein, which is the primary target of PSII photooxidative damage (Yoshioka and Yamamoto, 2011). A further enriched GO term of interest that highlights the efficiency of our GWAS in identifying genes involved in PSII activity is the ‘calmodulin binding’ molecular function (Fig. 6B). Calcium is an essential cofactor for the oxygen evolving complex of PSII that catalyses the oxidation of water (Barry *et al.*, 2005; Wang *et al.*, 2019), thus it is logical that predicted calmodulins (calcium-binding proteins) may be enriched in our candidate genes. Further support is lent to this from studies that have demonstrated that exogenous application of Ca^2+^ can stabilize PSII activity under heat stress (Tiwari *et al.*, 2019; Zheng *et al.*, 2022), thereby highlighting the importance of Ca^2+^ homeostasis for PHT, potentially achieved through calmodulin-mediated Ca^2+^ signalling.

Across the 133 candidate genes, the GO term most enriched was that associated with salicylic acid (SA) biosynthesis (Fig. 6A). SA has been well demonstrated to play a role in influencing the response of plants to heat stress, where it is best characterized by inducing antioxidant activity (Dat *et al.*, 1998; Nazar *et al.*, 2011; Khan *et al.*, 2013; Jahan *et al.*, 2019; Janda *et al.*, 2020). Antioxidant enzymes can protect PSII from damage due to ROS (Das and Roychoudhury (2014)). There is evidence suggesting that during rapid stress events, SA accumulation can have an alleviating effect on PSII photoinhibition. For example, Chen *et al.* (2020) demonstrated that under high light stress, SA accumulation increased photoprotection in Arabidopsis by enhancing the phosphorylation of the D1 and D2 PSII proteins and by reducing the rate of disassembly of the PSII–LHCII super complexes. The same authors have also shown that SA has a similar photoprotective role in wheat seedlings (Chen *et al.*, 2016).

Additionally, enriched GO terms highlight the potential of genes with defence roles regulating variation in *T*_crit_ and *T*_50_ (Fig. 6A). PSII is important for plant immunity because of its role in producing ROS, which can be important retrograde signalling molecules for coordinating defence responses (Järvi *et al.*, 2016; Foyer & Hanke, 2022). Consequently, genes involved in regulating ROS production to protect PSII during heat stress may additionally have roles in the signalling pathways associated with plant immunity. Indeed, disrupting chloroplastic function has been shown to impair resistance in wheat to *Septoria* leaf blotch (Lee *et al.*, 2015), where resistance to this end is associated with photoprotection (Ajigboye *et al.*, 2021).

We have confidence in our GO enrichment approach for validating our GWAS because of the identified and discussed terms. Additionally, we believe it is a valid approach in this instance because of the number of QTLs identified. Since we identified more than 10 QTLs for *T*_crit_ and *T*_50_ and inputted associated genes into the GO enrichment analyses we would expect some enrichment in genes involved in PSII activity. This would not be the case if we had identified only a few (~1–5 QTLs). Indeed, the number of genes associated with the enriched terms is small and consistent with the number of identified QTLs, but the fold enrichment and the significance attached to them is high (Fig. 6).

### Promising candidate genes for the development of heat tolerance in rice

Our approach for narrowing down the candidate genes (Fig. 1, Materials and methods) identified 30 genes for which we have high confidence in their role in PSII activity and/or heat tolerance (Table 3), and we highlight the most promising of these below.

We identified two genes whose Arabidopsis homologues are known to play essential roles in chloroplast development (Table 3), namely *DELAYED GREENING 1* (*DG1*) and *ALBINO OR PALE GREEN 3* (*APG3*). Knockout mutants of these two genes exhibit striking phenotypes. *dg1* mutant seedlings exhibit initially pale young leaves that gradually green to wild type levels (Chi *et al.*, 2008) whilst *apg3* mutants lack chlorophyll pigments and cannot photosynthesize (Motohashi *et al.*, 2007). Both genes appear to encode proteins involved in the formation of thylakoid membranes. The location of PSII within the thylakoid membrane further highlights the role these genes likely play in the activity of PSII where stable thylakoid complex assembly and maintenance will play an important role in heat tolerance. Furthermore, OsDG1 exhibits a 2-fold increase in expression in response to 42 °C heat stress in IR64 seedlings (Sharma *et al.*, 2021). Mutations in *AtDG1* have also been shown to result in temperature sensitivity and reduced *F*_v_/*F*_m_ at high temperatures relative to wild type, where the same phenotype is not observed under optimal growing temperatures (Sun *et al.*, 2020); here DG1 appears to be important for regulating chloroplastic mRNA editing at elevated temperatures.

We additionally identified several other genes with demonstrable roles in photosynthesis (Table 3). For example, *GOLGI LOCALIZED MONOSACCHRIDE TRANSPORTER 1* (*GST1*) encodes a protein that has been shown to play a role in sugar accumulation during abiotic stress (Cao *et al.*, 2011) and its Arabidopsis homologue, *pGlct*, encodes a protein involved in carbon partitioning, with mutants showing decreased photosynthesis (Cho *et al.*, 2011). pGlct has also been demonstrated to have a role in sugar (maltose) accumulation for conferring photoprotection of PSII (Kaplan and Guy, 2005). A further identified photosynthesis-related gene of interest is *SPOTTED LESSION 40* (*SPL40*, Table 3). SPL40 appears to be critical in activating SA signalling pathways and *spl40* mutants show hypersensitivity to light and a compromise in ROS homeostasis. This is associated with a downregulation in the expression of photosynthesis-associated genes and a reduction in chlorophyll content (Sathe *et al.*, 2019).

*Calmodulin-Like Protein Gene 21* (*CML21*) was identified within the Os-Tcrit-5 QTL. In Arabidopsis, CML21 functions as a calcium sensor coordinating Ca^2+^ signalling (McCormack and Braam, 2003), highlighting a potential role in Ca^2+^ homeostasis for protecting PSII. Further to this, the study of Aleynova *et al.* (2020) showed in grapevine that the native CML21 is differentially expressed in response to high temperatures. They also showed that heterologous overexpression of grapevine CML21 in Arabidopsis disrupted biomass accumulation in response to heat stress, highlighting the importance of functional CML21 activity.

Also associated with the Os-Tcrit-5 QTL were genes with sequence similarity to the Arabidopsis plasma-membrane localized receptor-like kinase FERONIA gene (Table 3; Supplementary Table S6). Recent evidence has pinpointed FERONIA in having a key role in regulating tolerance to photooxidative stress (L. Wang *et al.*, 2020; Shin *et al.*, 2021; Jing *et al.*, 2023). For example, Arabidopsis *fer* mutants are hugely light sensitive and demonstrate leaf bleaching when exposed to just moderate light intensities (Shin *et al.*, 2021). Here, *fer* mutants do not appear to be able to induce expression of key stress genes in response to light, such that ROS overaccumulate causing severe damage to PSII. In apple, overexpression of a native *FERONIA* gene markedly improved drought tolerance (Jing *et al.*, 2023). Here, *FERONIA* overexpression lines demonstrated significantly reduced photosystem damage and improved rates of photosynthesis compared with wild-type apple after 7 d of water withdrawal. The findings of these studies highlight the potential role of our identified FERONIA genes for improving photoprotection in response to heat in rice.

The recent study by Cubry *et al.* (2020) included the results of environmental-GWAS in *O. glaberrima*, where the authors performed GWAS on bioclimatic parameters specific to the point of origin of the same *O. glaberrima* accessions used in this present study. These bioclimatic parameters include those related to temperature. Since we are measuring temperature responses, we might expect to observe some overlap between environmental QTLs detected by Cubry *et al.* (2020) and our QTLs. To this end, we observed the colocalization (within 250 kb) of our Og-T50-8 QTL and a QTL detected for mean temperature-related parameters; 31 genes lie within this region (Supplementary Table S4) and include those with potential roles in conferring heat tolerance (Table 3). Os08g0135900, for example, is orthologous to Arabidopsis *TRYPTOPHAN SYNTHASE B SUBUNIT 1* (*TSB1*), whose protein has been shown to modulate tryptophan and abscisic acid biosynthesis to coordinate stress responses and growth in Arabidopsis (Liu *et al.*, 2022) and rice (Dharmawardhana *et al.*, 2013). Additionally, two genes in this region (Os08g0133000 and Os08g0133700) encode plant cysteine oxidases (Table 3; Supplementary Table S4). These enzymes are crucial in oxygen sensing and triggering various plant stress responses through the N-degron pathway to maintain cellular homeostasis in response to intracellular O_2_ and ROS accumulation (Holdsworth *et al.*, 2020; Heo *et al.*, 2021).

### Conclusion

With this study, we have adjusted our previous approach to measure the response of the maximum efficiency of PSII to increasing temperatures, such that it now truly represents a phenomics-like platform. The high estimates of heritability and broad genetic variation characterized through this platform highlight its utility for crop breeding, where *T*_crit_ and *T*_50_ could represent important covariates in rice selection models. Finally, we have assembled a list of high-confidence candidate genes representing targets for improving heat tolerance in rice.

## Supplementary data

The following supplementary data are available at *JXB* online:

Fig. S1. Schematic figure demonstrating the segmented modelling of the response of *F*_v._/*F*_m_ to temperature.

Fig. S2. Summary of results for GWAS for *T*_crit_ (*Oryza sativa*).

Fig. S3. Summary of results for GWAS for *T*_50_ (*Oryza sativa*).

Fig. S4. Summary of results for GWAS for *T*_50_ (*Oryza glaberrima*).

Fig. S5. Summary of results for GWAS for *T*_crit_ (*Oryza glaberrima*).

Fig. S6. Summary of results for GWAS for *m*_1_ and *m*_1_ (*Oryza sativa* and *Oryza glaberrima*).

Table S1. List of all accessions used in this study.

Table S2. All phenotypic data generated and used in this study.

Table S3. Pairwise trait interactions.

Table S4. GWAS results, significant SNPs.

Table S5. Genetic loci within the region on chromosome 8 between the Og-T50-8 QTL and eQTL (Cubry *et al*., 2020).

Table S6. Genes in linkage disequilibrium (LD>0.3) with significant SNPs within *O. sativa* QTLs.

Table S7. Gene ontology enrichment analysis of genes in LD with significant SNPs within *O. sativa* QTLs.

Table S8. Location of putative QTLs identified for *m*_1_ and *m*_2_ in *Oryza glaberrima* and *Oryza sativa*, according to EMMA GWAS model.

Table S9. Genome wide association results: significant SNPs associated with *m*_1_ and *m*_2_ across *Oryza glaberrima* and *Oryza sativa.*

Video S1. *F*_v_/*F*_m_ values of a series of samples at multiple temperatures throughout the experimental procedure.

erad239_suppl_Supplementary_Figures_S1-S6

erad239_suppl_Supplementary_Tables_S1-S9

erad239_suppl_Supplementary_Video_S1

## Data Availability

All data presented in this paper are available in the supplemental data.

## Abbreviations

BAAP: Bengal Assam Aus Panel
*F*_m_: maximum fluorescence
*F*_o_: minimum fluorescence
*F*_v_: variable fluorescence
*F*_v_/*F*_m_: maximum quantum yield of PSII
GO: Gene Ontology
GWAS: genome-wide association study
LD: linkage disequilibrium
PHT: photosynthetic heat tolerance
PSII: photosystem II
QTL: quantitative trait locus
SA: salicylic acid
SNP: single nucleotide polymorphism
*T*_50_: temperature at which PSII efficiency is 50% of its maximum
*T*_crit_: critical temperature point
