# Supplementary material for: Chlorophyll fluorescence-based high-throughput phenotyping facilitates the genetic dissection of photosynthetic heat tolerance in African (Oryza glaberrima) and Asian (Oryza sativa) rice
Source: J Exp Bot. 2023 Jun 22;74(17):5181–97. doi: 10.1093/jxb/erad239 (PMC10498015; doi:10.1093/jxb/erad239)
Supplement: erad239_suppl_Supplementary_Figures_S1-S6 [file erad239_suppl_supplementary_figures_s1-s6.pdf]

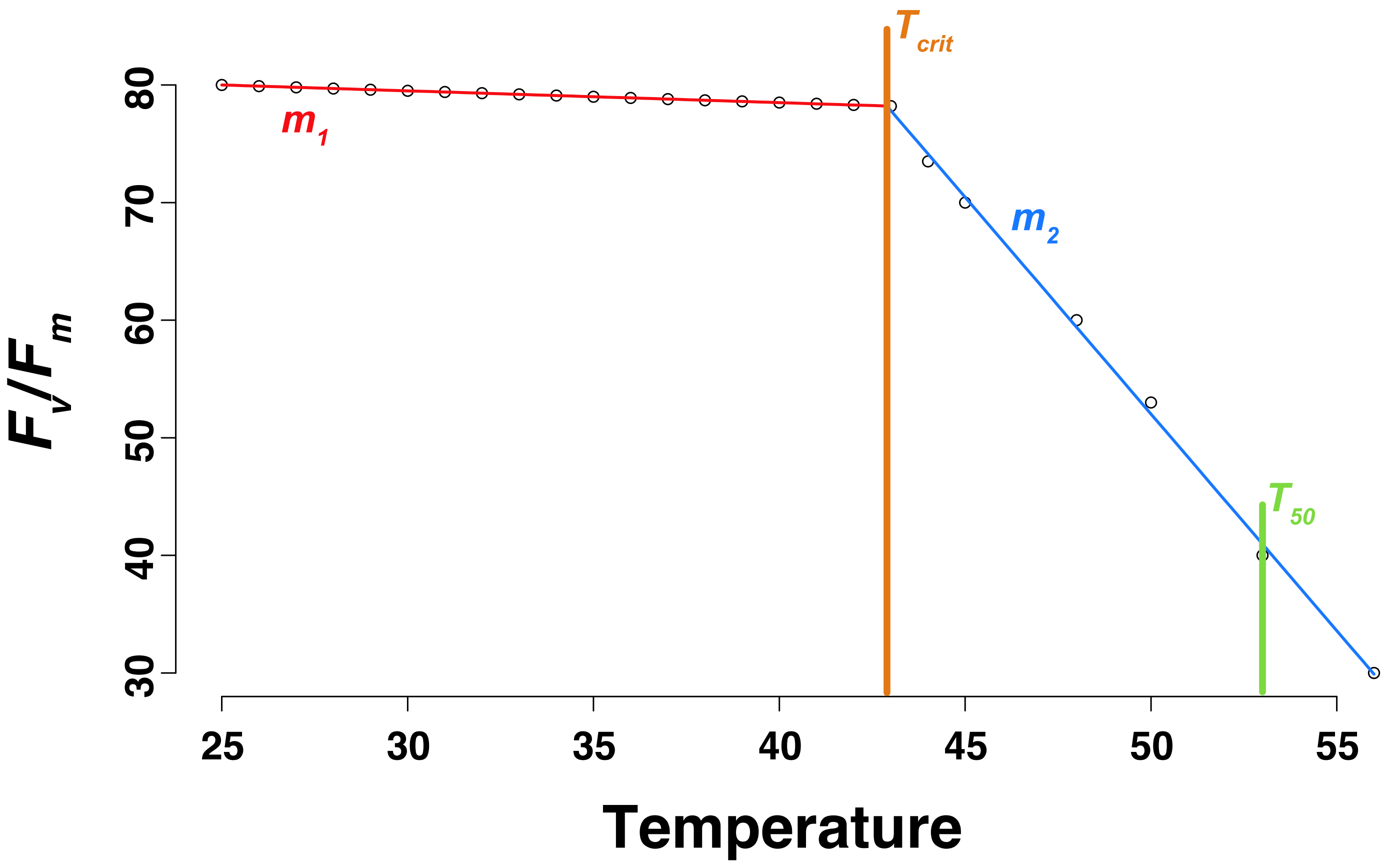

Supplemental Figure S1. Schematic figure demonstrating the segmented modelling of the response of F_v/F_m to temperature

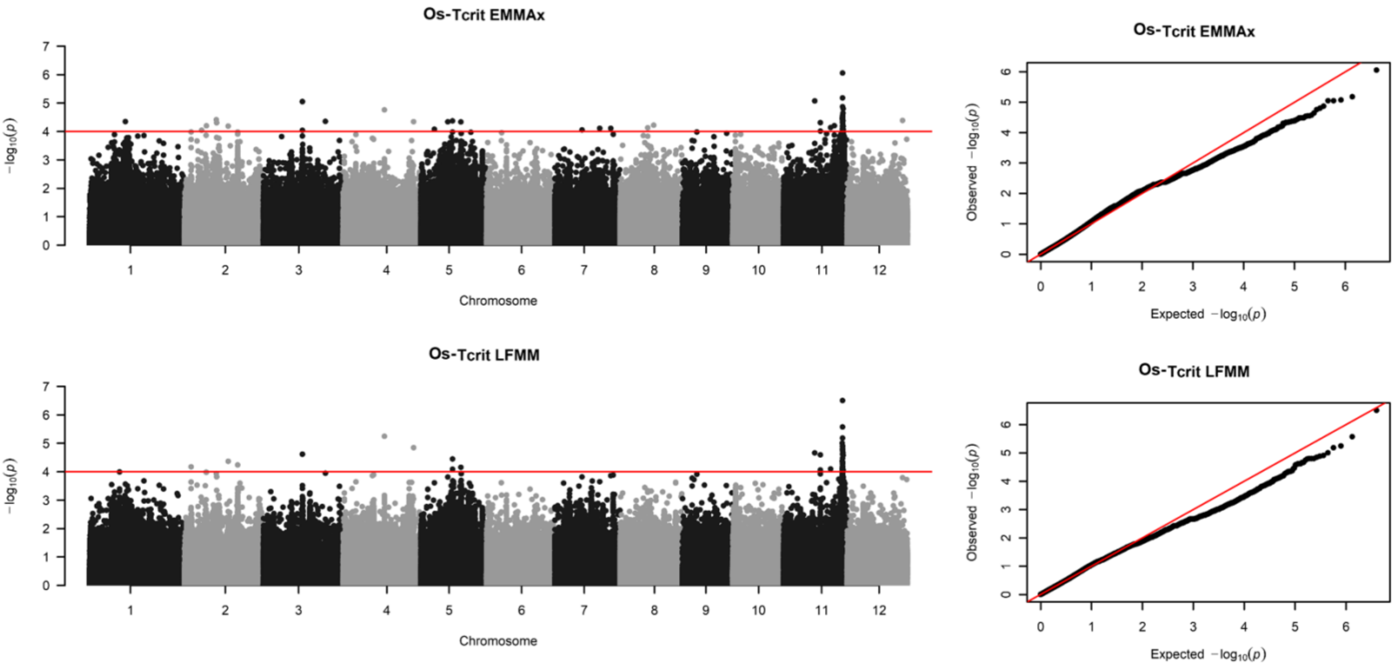

Supplemental Figure S2. Summary of results for GWAS for T_{crit} (*Oryza sativa*)

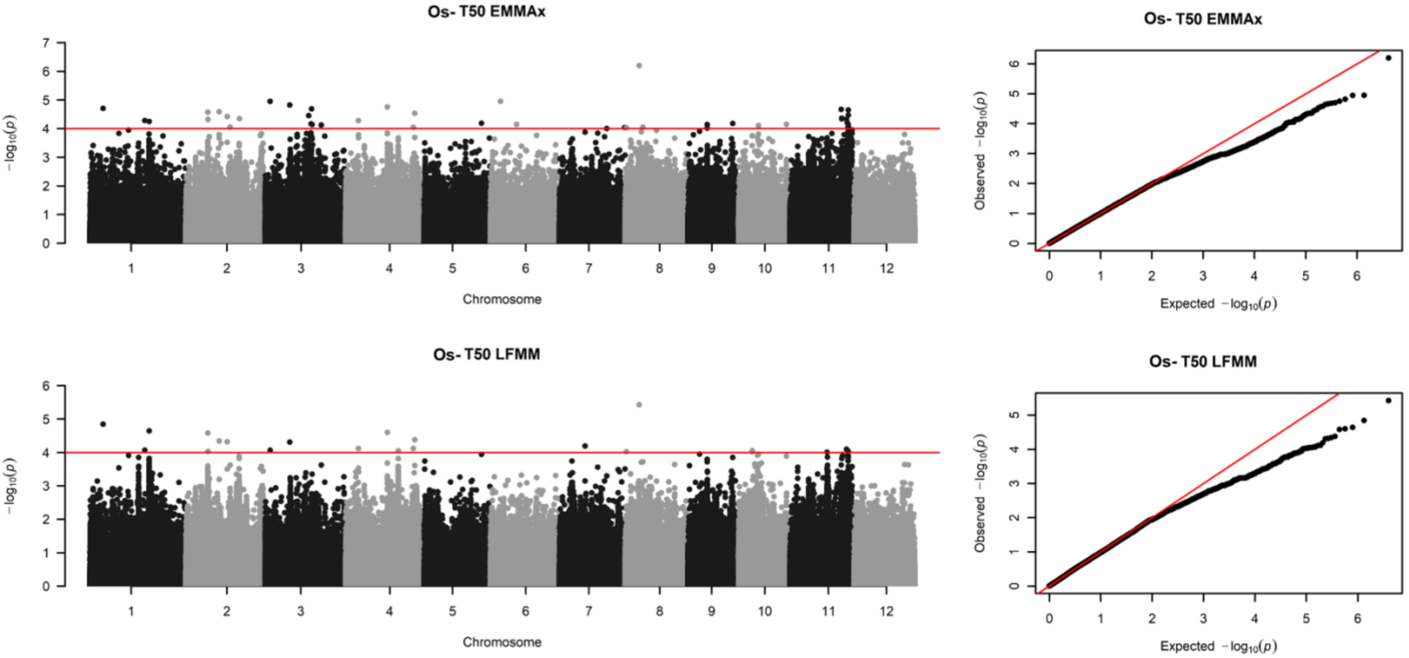

Supplemental Figure S3. Summary of results for GWAS for T_{50} (*Oryza sativa*)

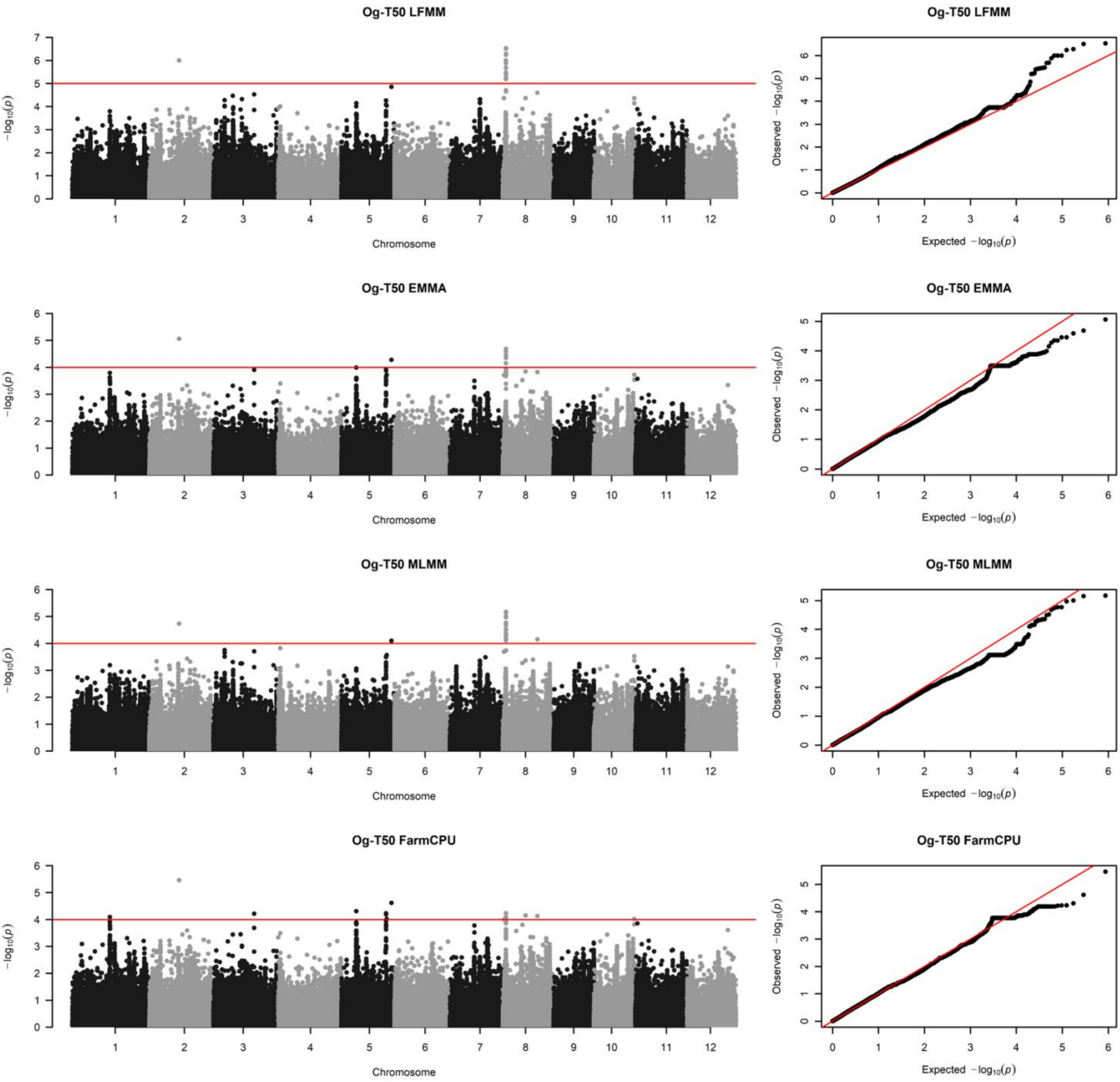

Supplemental Figure S4. Summary of results for GWAS for T_{50} (*Oryza glaberrima*)

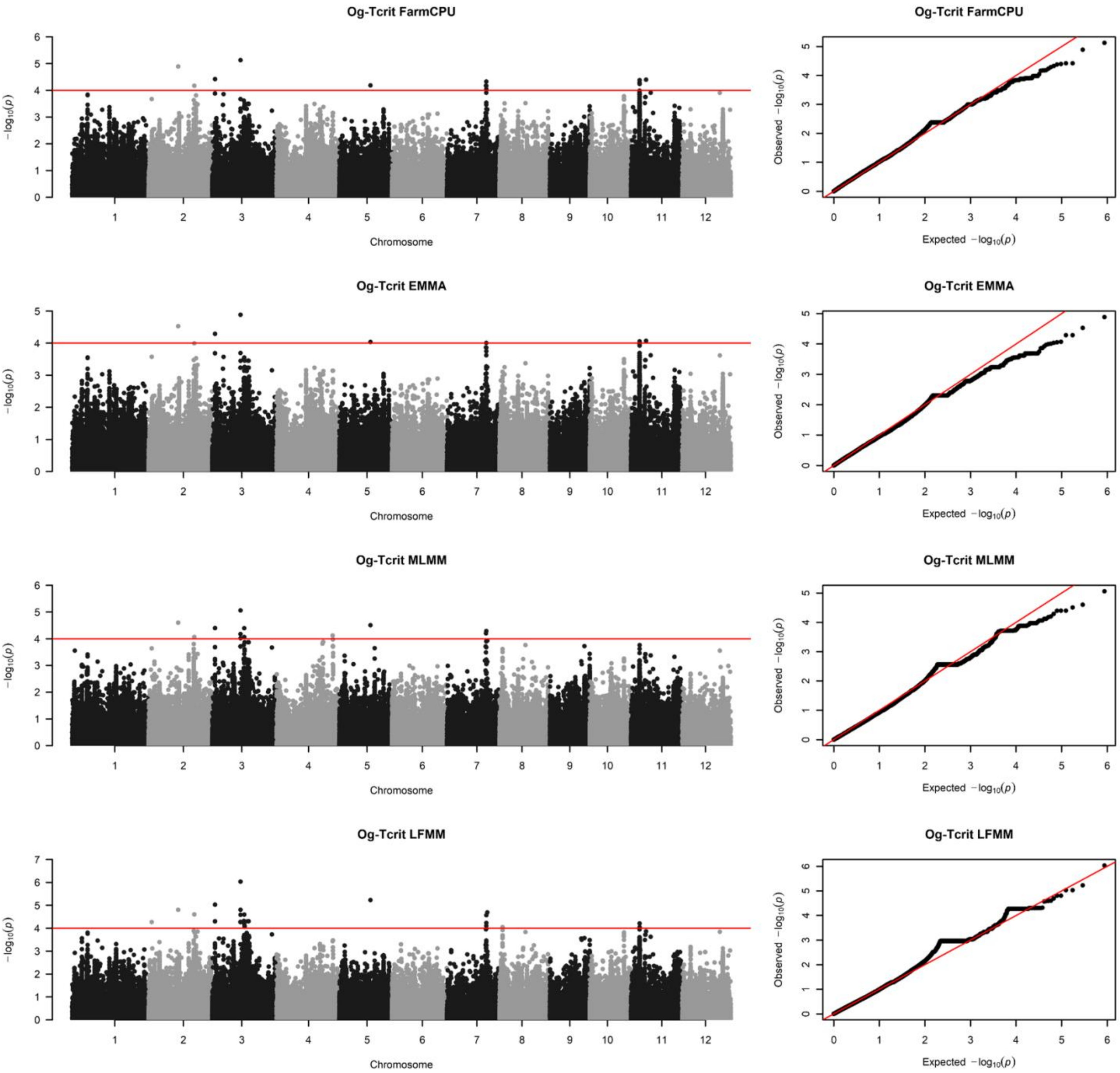

Supplemental Figure S5. Summary of results for GWAS for T_{crit} (*Oryza glaberrima*)

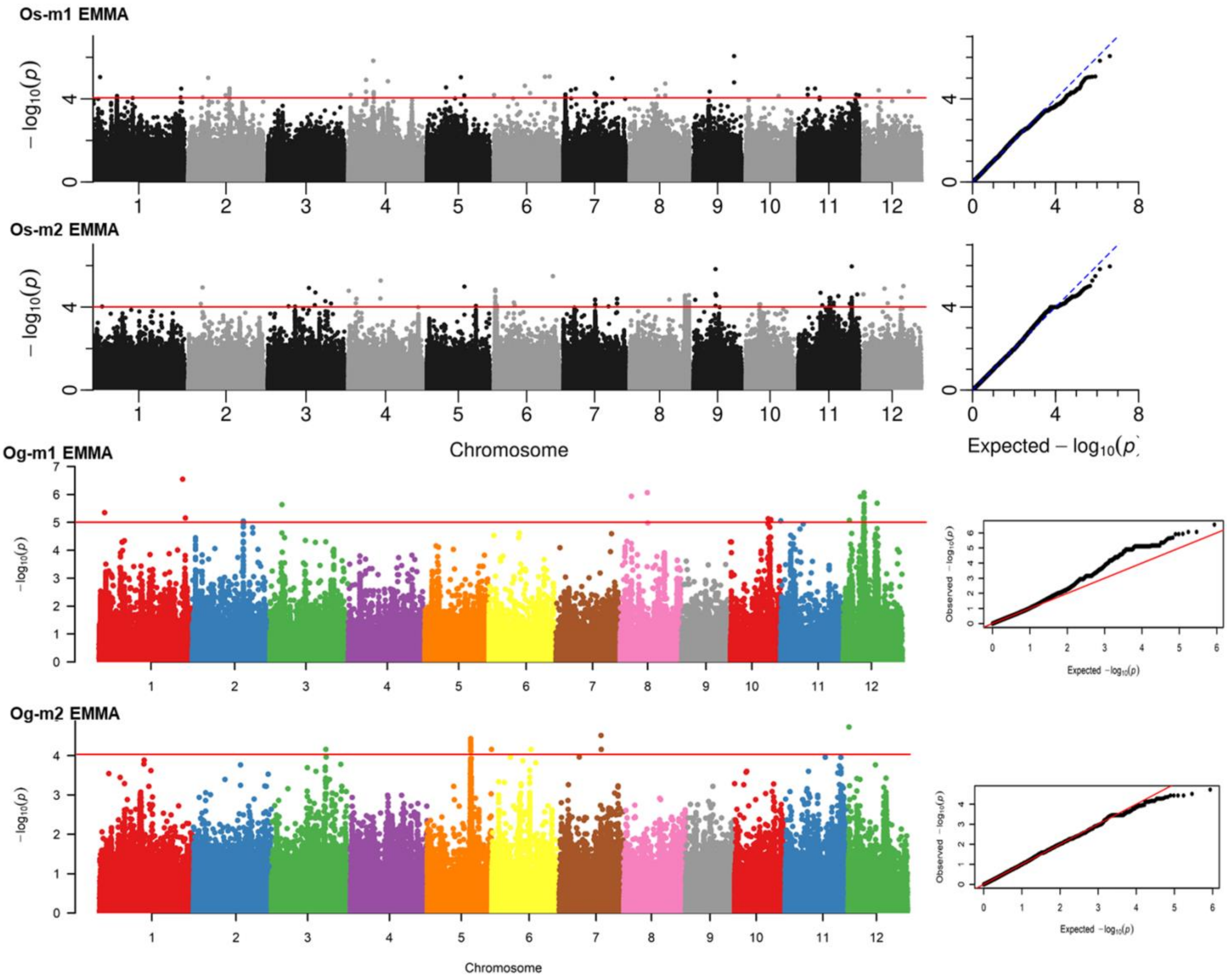

Supplemental Figure S6. Summary of results for GWAS for m_1 and m_1 (*Oryza sativa and Oryza glaberrima*)
